# Tracking of Tumor Cell–Derived Extracellular Vesicles *In Vivo* Reveals a Specific Distribution Pattern with Consecutive Biological Effects on Target Sites of Metastasis

**DOI:** 10.1007/s11307-020-01521-9

**Published:** 2020-07-31

**Authors:** Mirjam Gerwing, Vanessa Kocman, Miriam Stölting, Anne Helfen, Max Masthoff, Johannes Roth, Katarzyna Barczyk-Kahlert, Lilo Greune, M. Alexander Schmidt, Walter Heindel, Cornelius Faber, Simone König, Moritz Wildgruber, Michel Eisenblätter

**Affiliations:** 1 Institute of Clinical Radiology, University Hospital Münster, Münster, Germany; 2 Institute for Immunology, University Muenster, Muenster, Germany; 3 DFG Cluster of Excellence EXC 1003 ‘Cells in Motion’, Muenster, Germany; 4 ZMBE, Institute of Infectiology, University Münster, Muenster, Germany; 5 IZKF Core Unit Proteomics, University of Muenster, Muenster, Germany; 6 Klinik und Poliklinik für Radiologie, Klinikum der Universität München, Munich, Germany; 7 Department of Diagnostic and Interventional Radiology, University Hospital Freiburg, University of Freiburg Medical Center, Hugstetter St. 55, 79106 Freiburg im Breisgau, Germany

**Keywords:** Extracellular vesicles, Metastasis, Optical imaging, Proteomics

## Abstract

**Purpose:**

Extracellular vesicles, small vesicles carrying *inter alia* proteins, miRNA and RNA, are important mediators of intercellular communication. The purpose of this study was to assess the distribution of extracellular vesicles from highly malignant breast cancer and their subsequent effect on the immune cell infiltrate in target organs of metastasis.

**Procedures:**

Extracellular vesicles were isolated from the tissue culture supernatant of highly malignant 4T1 breast cancer cells or the serum of healthy BALB/c mice. The purity of the isolate was verified by electron microscopy and western blotting. Extracellular vesicles were additionally subjected to proteome analysis. After labeling with the fluorescent dye DiR, extracellular vesicles were injected into healthy BALB/c mice and their *in vivo* distribution was assessed using fluorescence reflectance imaging (FRI). Following *ex vivo* imaging of the organs, lung tissue samples were analyzed for extracellular vesicle-mediated changes of myeloid cells and T cell numbers, using flow cytometry. Proteome analysis revealed major differences in the cargo of tumor cell–derived *versus* extracellular vesicles from healthy serum.

**Results:**

In contrast to control extracellular vesicles, DiR-labeled extracellular vesicles from tumor cells preferentially accumulated in lung, liver, and spine. Subsequent flow cytometry of the immune cell composition of lung tissue samples revealed an increase of cytotoxic CD8+ T cells and a decrease of CD4+ T-helper cells as well as an increase in mature macrophages in response to tumor cell EV.

**Conclusions:**

In conclusion, distribution of tumor cell–derived extracellular vesicles follows a specific pattern and can be monitored, using dedicated imaging. Extracellular vesicles alter the immune cell composition in target organs of metastasis, using a specific proteome cargo.

**Electronic supplementary material:**

The online version of this article (10.1007/s11307-020-01521-9) contains supplementary material, which is available to authorized users.

## Introduction

Cancer cells rely on the recruitment and activation of various benign, physiological cells, most prominently immune cells, to facilitate and support growth, invasion, and finally metastasis. Mediators of the required cell-cell interaction have thus been a major focus of research, not only for understanding the process of metastatic spread but also for identification of biomarkers for disease stage, progression, and resistance mechanisms [1, 2].

Originally described as part of a waste disposal system, extracellular vesicles have more recently been identified as cell-cell communicators, shed ubiquitously by a variety of cell types [3]. Tumor cells exhibit an enhanced secretion of extracellular vesicles, which are of 50–150-nm diameter in size [3]. They contain *inter alia* (mi)RNA, proteins, and peptides and their individual content largely reflects the parental cell [4]. As the transport medium of a variety of molecules, extracellular vesicles are thought to contribute to metastatic spread *via* aid in induction of a premetastatic niche. This premetastatic niche forms as a tumor-supportive inflammatory environment, detectable in the target tissue before the establishment of metastases [5]. After fusion of multivesicular bodies with the cell membrane and release into the blood stream, extracellular vesicles distribute systemically, where they exert their effects in one of three different ways: (1) binding of extracellular vesicles to a membrane protein of the target cell activates a dedicated signal pathway inside the cell; (2) a protease in the extracellular matrix cleaves the extracellular vesicle membrane proteins, which afterwards bind to receptors on the cell membrane, activating a signaling pathway; (3) the extracellular vesicle membrane fuses with the target cell membrane, causing nonselective release of its content [6].

For extracellular vesicles to effectively contribute to premetastatic niche formation, their distribution would have to follow a specific pattern exhibiting a specific effect on the immune cell population in the target tissue of metastasis. These extracellular vesicles also induce a high vascular permeability and altered bone marrow progenitors towards a pro-angiogenic phenotype [7]. Extracellular vesicles derived from pancreatic ductal adenocarcinoma cells proved to induce liver premetastatic niche formation in naïve mice and increase liver metastatic burden. In the liver, an enhanced recruitment of bone marrow–derived macrophages was provoked [8]. It has also been shown that treatment with chemotherapy leads to a selection of extracellular vesicles which induce endothelial cell activation and monocyte expansion in the premetastatic niche in the lungs to facilitate lung metastases in a breast cancer model [9].

In this study, we want to use *in vivo* tracking of tumor-derived exosomes by molecular imaging for further elucidation of the potential of tumor-derived extracellular vesicles as mediators of premetastatic tissue priming. In a model of metastatic breast cancer, we want to compare the dynamic *in vivo* distribution of extracellular vesicles from different origin (tumor cell–derived *vs* non-tumorous extracellular vesicles) and examine their biological effects in target organs of metastasis as well as the respective distribution pattern.

We want to assess whether it is the biochemical content of extracellular vesicles alone that holds the key to premetastatic tissue priming or if the systemic distribution and subsequent uptake of extracellular vesicles also follows a specific pattern potentially explaining the organotropism of specific metastatic spread.

## Material and Methods

### Experimental Design

In a first step, the isolation of extracellular vesicles from both, mouse serum and cell culture supernatant, was set up, adapting established protocols [10]. The isolated extracellular vesicles were first analyzed for their purity and subsequent exclusion of concomitantly isolated particles. The proteome content of extracellular vesicles from tumor cells as well as healthy mouse serum was analyzed using mass spectrometry. Before *in vivo* imaging experiments, feasibility and effectivity of fluorescence labelling of extracellular vesicles of different origin was confirmed. In first *in vivo* experiments, we assessed the dynamics of extracellular vesicle distribution and local accumulation, comparing two groups: the control group was injected with extracellular vesicles isolated from serum of healthy BALB/c mice and the experimental group received extracellular vesicles from the cell culture supernatant of highly malignant 4T1 tumor cells.

After the *in vivo* assessment, organs were harvested and subjected to *ex vivo* imaging for a biodistribution analysis. The lungs were subsequently prepared for flow cytrometric analysis of the cellular composition with regard to the immune cell infiltration. An overview over the experimental design of this study is given in Supplementary Figure 1.

### Animals

All animal experiments in this study have been approved by the responsible authorities (reference of local government approval ID 81-02.04.2017-A431). All applicable institutional and/or national guidelines for the care and use of animals were followed. Forty-five female BALB/c mice (age 8–12 weeks), sourced from Charles River, were used for the experiments. Mice were dehaired using a razor and depilatory cream on back and bottom the day before start of the experiment to avoid artifacts in the fluorescence reflectance imaging (FRI) scan.

### Cell Culture

Murine breast cancer cell line 4T1 was cultured in RPMI medium (Sigma-Aldrich, St. Louis, Missouri), supplemented with XerumFree (TNCBio, Eindhoven, Netherlands) instead of extracellular vesicle-containing fetal bovine serum. Cells were grown until a confluency of approximately 80 % and cell culture supernatant was collected for extracellular vesicles isolation after 48 h of incubation.

### Extracellular Vesicles Isolation

Extracellular vesicles were isolated from the cell culture supernatant of 4T1 tumor cells (experimental group) as well as from the serum of healthy BALB/c mice (control group). Serum of healthy BALB/c mice was extracted from whole blood samples (1–1.5 ml), obtained *via* direct heart puncture of mice in deep prilocaine/ketamine anesthesia. Isolation of extracellular vesicles was performed as described previously for the isolation of extracellular vesicles from human plasma [10]. Briefly, the cell culture supernatant or murine serum was freed of debris with differential centrifugation, before ultrafiltration with a 0.22-μm filter. Size exclusion chromatography on a Sepharose 2B column was followed by ultracentrifugation at 105.000*g* for 2 h at 4 °C (see Fig. 2). The extracellular vesicle pellet was resuspended in PBS before further usage.

### Quantification of Extracellular Vesicles

Quantification of extracellular vesicles based on proteins was performed in a 96-well plate using the Pierce Bicinchoninic acid assay (BCA) protein assay kit (Thermo Fisher, Waltham, Massachusetts) according to the manufacturer’s protocol. A Spark Tecan Reader (Tecan Trading AG, Maennedorf, Switzerland) was used for the read out of the plate. The results were used to determine the amount of extracellular vesicles needed for injection (100 μg). Quantification of extracellular vesicles based on the number of particles, as well as size determination, was achieved using a NanoSight system (Malvern Instruments, Malvern, UK) with a green 532-nm laser and an Andor CCD camera.

### Western Blotting

Western blotting with specific extracellular vesicles markers was used to ensure that the isolate indeed consisted of extracellular vesicles, whereas other cell organelles like golgi were not present. Western blotting was performed as described previously [11]. In brief, 20 μg of extracellular vesicles was mixed with 4-μl lane marker, boiled at 95 °C for 5 min. The samples were quickly cooled down by placing them on ice, spun down and subjected to SDS-PAGE. Proteins were transferred from the gel to a PVDF membrane (Carl Roth, Karlsruhe, Germany) using a semidry transfer method. Afterwards, the membrane was blocked with 5 % BSA-TBS-T (0.2 M Tris, 0.15 M NaCl and Tween20) and then exposed to primary antibody solution (GM130 6170823, BD Biosciences, Franklin Lakes, New Jersey; TSG101 sc-7964 / CD81 sc-23,962, Santa Cruz, Dallas, Texas), overnight at 4 °C. After a washing step, the membranes were incubated with the corresponding secondary antibody for 1 h at room temperature. After a second washing step, the resulting chemiluminescence signals of the substrate, converted by the bound HRP-conjugated secondary antibody, were detected with Pierce ECL Western Blotting Substrate (Thermo Fisher Scientific, Waltham, Massachusetts) using the FRI system.

### Electron Microscopy

Electron microscopy enabled visualization of the extracellular vesicles to ensure that they are of typical shape and size, and are intact after isolation. Negative staining was used to visualize the isolated extracellular vesicles. Extracellular vesicles (5 μl) were sedimented on Formvar-coated, carbon-sputtered cupper-grids.

After negative staining with 1 % phosphotungstic acid for 2 min, the samples were dried on filter paper and afterwards analyzed at 80 kV on a Tecnai 12 electron microscope (FEI, Hillsboro, Oregon). Images of selected areas were documented with Veleta 4 k CCD camera (emsis, Muenster, Germany).

### Proteome Analysis of Extracellular Vesicles Content

Three replicate samples were prepared of each extracellular vesicles. Samples (50 μg protein) were lysed in 8 M urea, 100 mM Tris, 0.5 % (*w*/*v*) SDS, 10 mM Tris(2-carboxyethyl)phosphin (TCEP) with ultrasonic treatment (15 min) and centrifuged (4 °C, 30 min, 20.000×*g*). Proteins were prepared for mass spectrometry-based expression analysis with Synapt G2 Si coupled to M-Class nanoUPLC (Waters Corporation, Milford, Massachusetts) as previously described [12]. Briefly, proteins were reduced, alkylated, digested using trypsin, and injected into the instrument at 1 μg/μl (3 μl injections) after evaluating the signal intensities in diagnostic runs. Measurements were performed in replicate and data were analyzed using Progenesis QI for proteomics software (Nonlinear Diagnostics) and the UniProt database for *Mus musculus*. For subsequent considerations, the output was restricted to proteins with minimum two peptide hits, a fold value > 2 and an ANOVA value *p* ≤ 0.05. The heatmap was created using Heatmapper software [13]. Pathway and network analyses were performed using PantherDB (University of Southern California, USA) and Cytoscope software [14].

### Extracellular Vesicles Labeling and Injection

For labeling of the extracellular vesicles, 100 μg of extracellular vesicles was incubated with 2 μM of DiR (diluted with PBS) for 15 min at room temperature. Afterwards, ultracentrifugation for 90 min at 105.000*g* pelleted the labeled extracellular vesicles, before resuspension in PBS and preparation for further use. In order to secure a comparable signal intensity of the labeled extracellular vesicles between the tumor cell–derived extracellular vesicles and the control extracellular vesicles from healthy mice, *in vitro* dilution experiments were done. One hundred micrograms, 50 μg, 25 μg, and 10 μg of DiR-labeled exosomes were resuspended in PBS and pipetted into a 96-well plate, before measurement of signal intensities using the FRI system.

For the *in vivo* experiments, 100 μg of extracellular vesicles was labeled and resuspended in 100 μl of PBS. This amount was chosen to have a sufficient amount for the induction of changes in the immune cell composition of the lung. Injection in the tail vein was performed under isoflurane anesthesia (2 % isoflurane plus 0.9 l O_2_), as were the *in vivo* scans.

### *In Vivo* Imaging

Before injection of the extracellular vesicles, a baseline FRI scan was performed to check for individual autofluorescence signals. Imaging was then conducted at different time points after extracellular vesicle injection for the kinetics experiment (30 min, 3 h, 6 h, 12 h, 24 h, 48 h) and after 24 h to assess accumulation of the extracellular vesicles. For *in vivo* optical imaging, a fluorescence reflectance imaging (FRI) system (Bruker BioSpin, Billerica, Massachusetts) was used. Excitation wavelength was adapted to DiR (750 nm), and the resulting emission was recorded at 780 nm, using a filter equipped, high-sensitive charge-coupled device camera. Signal acquisition time was ten minutes for fluorescence images. White-light and X-ray images were acquired for anatomic correlation. After *in vivo* imaging, the mice were sacrificed and the parenchymatous organs harvested for *ex vivo* biodistribution analysis. The organs were placed on a petri dish and additional *ex vivo* FRI imaging performed similarly to the *in vivo* imaging.

For the analysis, a region of interest (ROI) was placed around the organs, similarly for the *in vivo* and *ex vivo* scans. FRI data were presented as mean intensity, organ intensity of *ex vivo* organs was normalized to the signal intensity of the muscle, resulting in an organ/muscle ratio. The fluorescence signal is measured in arbitrary units (a.u.). The lung was harvested and prepared for flow cytometry analysis.

### Flow Cytometric Analyses

For flow cytometric analyses, single-cell suspensions were produced from lung tissue. The cell content was determined and the cells stained with the antibodies and their corresponding isotype controls: Cd11b-PB (#101224, BioLegend, San Diego, California) and isotype control rat IgG2b,κ – PB (#400627, BioLegend, San Diego, California), Ly6C-PE (#128007, BioLegend, San Diego, California) and isotype control rat IgG2c, κ – PE (#400707, BioLegend, San Diego, California), F4/80-APC (#123116, BioLegend, San Diego, California) and isotype control rat IgG2a, κ – APC (#17–4321-81, eBioscience, San Diego, California), CD4-APC (#100412, BioLegend, San Diego, California) and isotype control rat IgG2b, κ – APC (#553991, BD Biosciences, Franklin Lakes, New Jersey), CD8a-FITC (#100706, BioLegend, San Diego, California) and isotype control rat IgG2a, κ – FITC (#553929, BD Biosciences, San Diego, California), CD3-PB (#100213, BioLegend, San Diego, California) and isotype control rat IgG2b, κ – PB (#400627, BioLegend, San Diego, California). Flow cytometry data was gated according to size and granularity to exclude cell detritus; isotype controls served for adjustment of individual flow cytometry measurements. All flow cytometry measurements were conducted using a fluorescence-activated cell sorting (FACS) Calibur system (Becton Dickinson, Franklin Lakes, New Jersey) and analyzed using the FlowJo software (FlowJo LLC, Ashland, Oregon). Data were presented as event frequency reduced by the individual isotype control to exclude unspecific staining.

### Statistical Analysis

Data were analyzed using the GraphPad Prism software (version 8; GraphPad Software Inc., San Diego, California). A Kolmogorow-Smirnow test ensured a normalized distribution pattern of our data, so differences in imaging between the results from the control (extracellular vesicles from the serum of healthy mice) and experimental group (tumor cell–derived extracellular vesicles) were evaluated using Student’s *t* test, for the fluorescent signal, as well as for differences in the immune cells. A *p* value below 0.05 was considered significant.

## Results

### *In Vitro* Analysis of Extracellular Vesicles

**Fig. 1:**
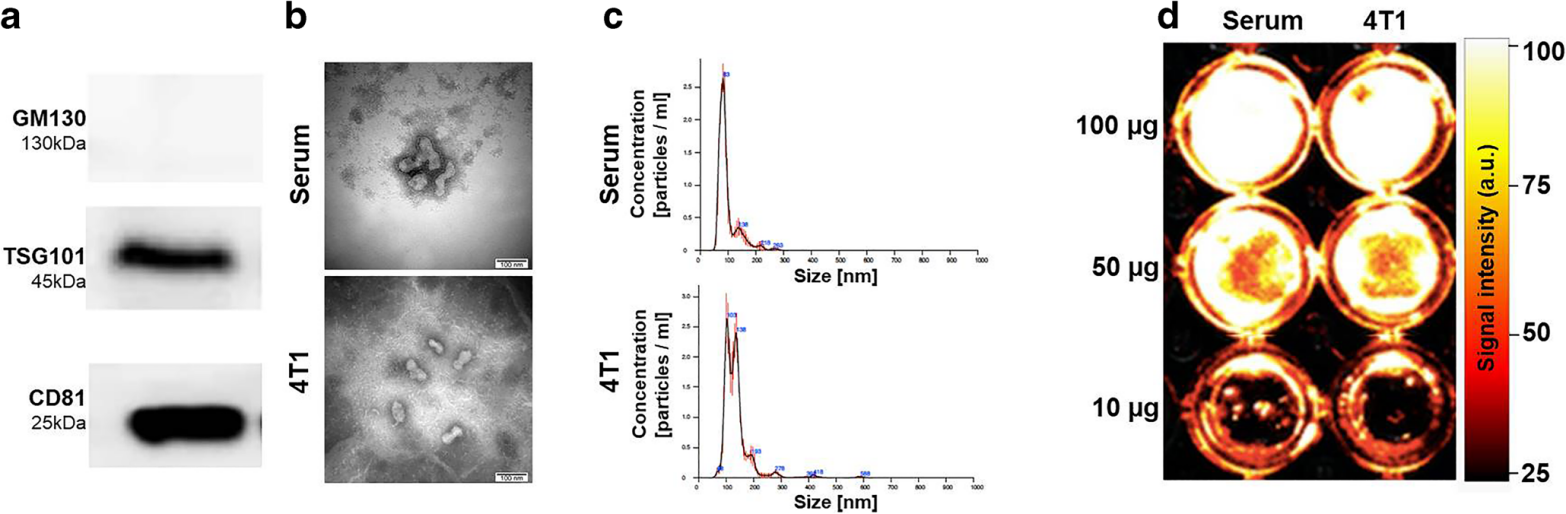
Analysis of isolated extracellular vesicles. **a** Western blotting shows positive bands for the extracellular vesicle markers TSG101 and CD81, whereas the golgi marker GM130 is negative. GAPDH is a housekeeping gene and quality control for the western blot. **b** Electron microscopy of extracellular vesicles, the left panel visualizes extracellular vesicles from the serum of healthy mice, the right panel displays tumor cell–derived extracellular vesicles. **c** NanoSight measurements of serum extracellular vesicles in the upper chart, tumor–derived extracellular vesicles in the lower chart reveal a slightly larger size of tumor cell-derived extracellular vesicles. **d** Dilution range of DiR-labeled extracellular vesicles, serum-derived extracellular vesicles on the left, tumor cell–derived extracellular vesicles on the right show comparable signal intensities for labeled extracellular vesicles of the two different origins. The excitation wavelength was set to 750 nm, emission was recorded at 780 nm.

The isolation of extracellular vesicles was successful and the resulting suspension was virtually free of other organelles or cell debris, as confirmed by electron microscopy and western blotting (Fig. 1a, b). Analysis of the extracellular vesicle size with NanoSight revealed a mean size of 135.7 ± 50.6 nm for the tumor cell–derived extracellular vesicles from cell culture supernatant of 4T1 cells, whereas extracellular vesicles from the serum of healthy mice were smaller, with a mean size of 97.3 ± 36.2 nm (Fig. 1c). This is in line with studies that isolated extracellular vesicles from the serum of C57BL/6 mice with an average size of 91 nm [15] and extracellular vesicles from the cell culture supernatant of pancreas carcinoma cells (MIAPaCa) with a size range from 120.07 to 132.7 nm [16].

### Proteome Analysis of Extracellular Vesicles

**Fig. 2:**
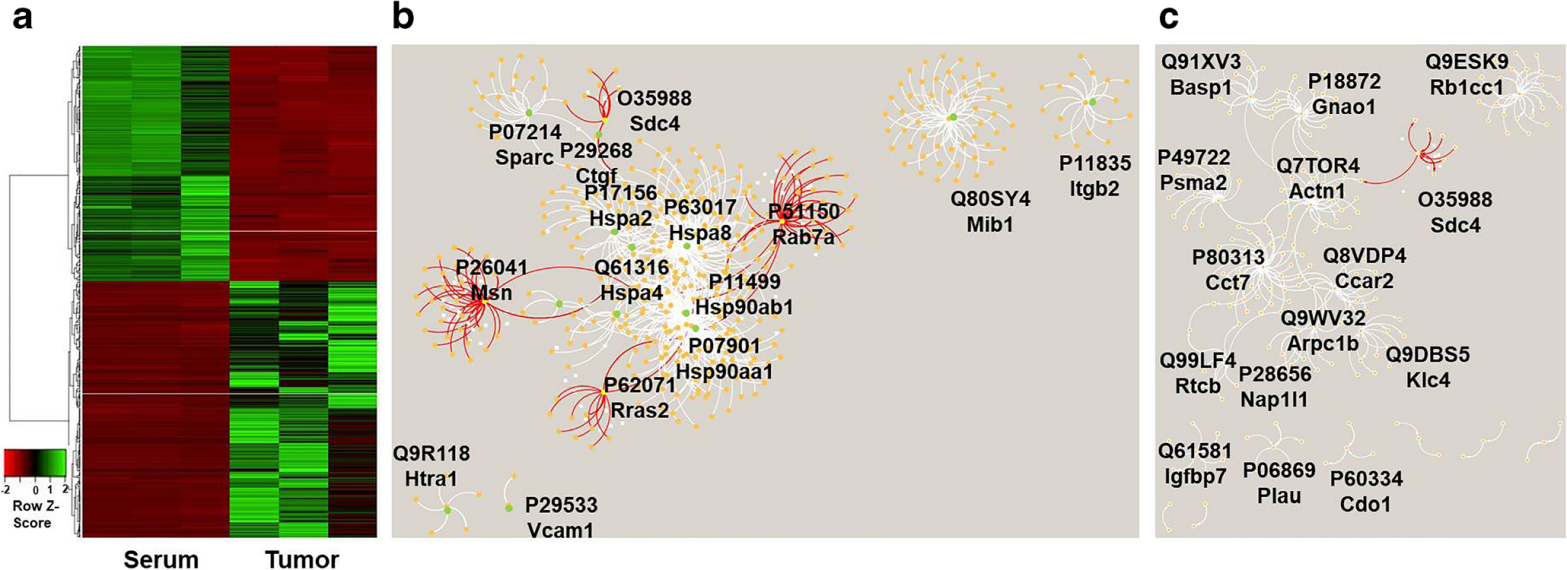
Visualization of mass spectrometry–derived protein data. **a** Heatmap of shortlisted entries for biological replicates. **b**, **c** Cytoscape analysis of selected proteins. Cluster nodes are labeled. **b** Proteins more abundant in tumor cell–derived extracellular vesicles with a fold value > 100 (Supplementary Table S8). C) Proteins of interest such as malignancy markers and ECM proteins (Supplementary Table S3). Nodes for moesin, syndecan and Ras-related proteins Rab7a and Rras2 are marked in red.

Proteome analysis was performed to identify differences in the extracellular vesicle cargo that might be responsible for potential differences in distribution and effects. The comparison of mass spectrometry–based protein analyses of tumor cell–derived and serum-derived extracellular vesicles revealed significant differences of their respective protein content (see heatmap in Fig. 2a). One biological replicate of serum-derived extracellular vesicles presented as outlier and was removed from statistical analysis. A total of 1762 proteins were detected which were short-listed based on their expression strength and ANOVA probability values (for data and principal component analysis, see Supplementary Tables S1 and S2, for gene ontology analysis see Supplementary Tables S4-S7). The known interactions of proteins, which were present 100-fold more in tumor cell extracellular vesicles as compared to extracellular vesicles from healthy serum, were visualized using network analysis (Fig. 2b, see Supplementary Table S8 for gene ontology analysis, see also analysis for 10-fold changes in Supplementary Table S9). Briefly, tumor cell–derived extracellular vesicles contained a substantially different set of proteins compared to extracellular vesicles isolated from healthy serum (Supplementary Tables S1 and S2). Representatives of more protein classes were present in tumor cell–derived extracellular vesicles at 100-fold higher concentration compared to control affecting about twice as many metabolic pathways as *vice versa* (Supplementary Table S8). Many proteins were, however, not present in such drastic concentration differences albeit at fold changes > 2. Among them were a number of highly interesting proteins in the context of cancer (Supplementary Table S3). Their network is illustrated in Fig. 2c. It shows the many interactions of heat shock factors and their direct involvement with Msn, Rab7a, Rras2, Sparc, and syndecan-4, whereas for Mib1 and Itgb2 no known connections to this network exist yet.

### Biodistribution of Extracellular Vesicles

As a next step, after analysis of the differing cargo between the two groups of extracellular vesicles, their *in vivo* biodistribution was assessed. *In vitro*, equal amounts of extracellular vesicles from the cell culture supernatant of tumor cells and the serum of healthy mice yielded a comparable fluorescence signal (mean intensities serum–derived *vs.* tumor cell–derived: 320.9 ± 129 *vs.* 338.6 ± 149; 210.8 ± 54.1 *vs.* 205.1 ± 135; 98.5 ± 74 *vs.* 100.9 ± 23.7; 31.1 ± 17.1 *vs.* 27.6 ± 21.5; Fig. 1d).

**Fig. 3:**
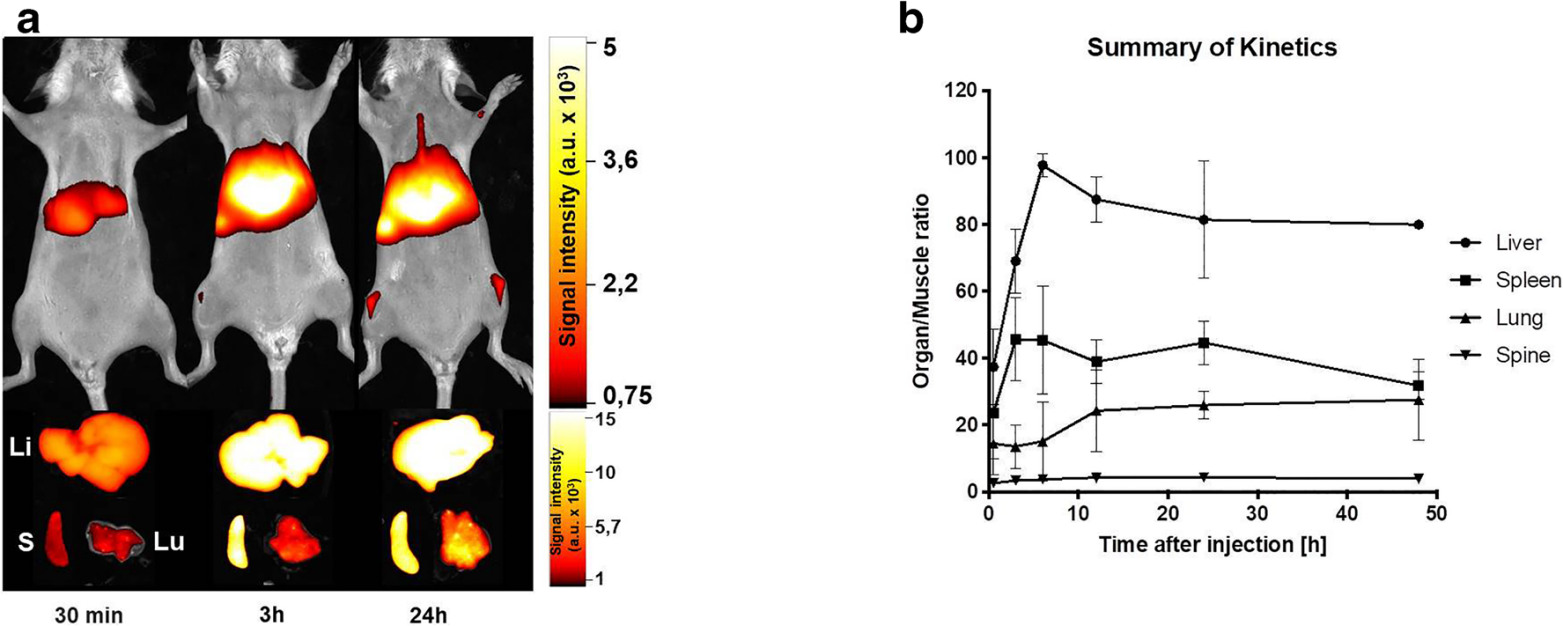
Kinetics of extracellular vesicles accumulation visualize a peak of extracellular vesicle accumulation in the liver after 6 h, followed by a steady decrease, whereas the accumulation in the lung peaks after twelve hours and remains constant for the first 48 h. **a** Exemplary *in vivo* (upper panel) and *ex vivo* (lower panel) images of mice 0, 5, 3, and 24 h after the injection of DiR-labeled tumor cell–derived extracellular vesicles. Li = liver, S = spleen, Lu = lung. **b** Summary of three independent experiments in the organs of interest. Values are the ratio of mean signal intensities organ to muscle.

In the kinetic study, DiR-labeled tumor cell–derived extracellular vesicles initially exhibited an unspecific perfusion of all organs after 30 min, before accumulation in target organs of metastasis. This accumulation was highest after six hours in the liver, before the signal intensity started to fade off again. In the lung, accumulation happened slower, with a steady increase of the signal intensity up to our last measurement at 48 h after injection (Fig. 3).

**Fig. 4:**
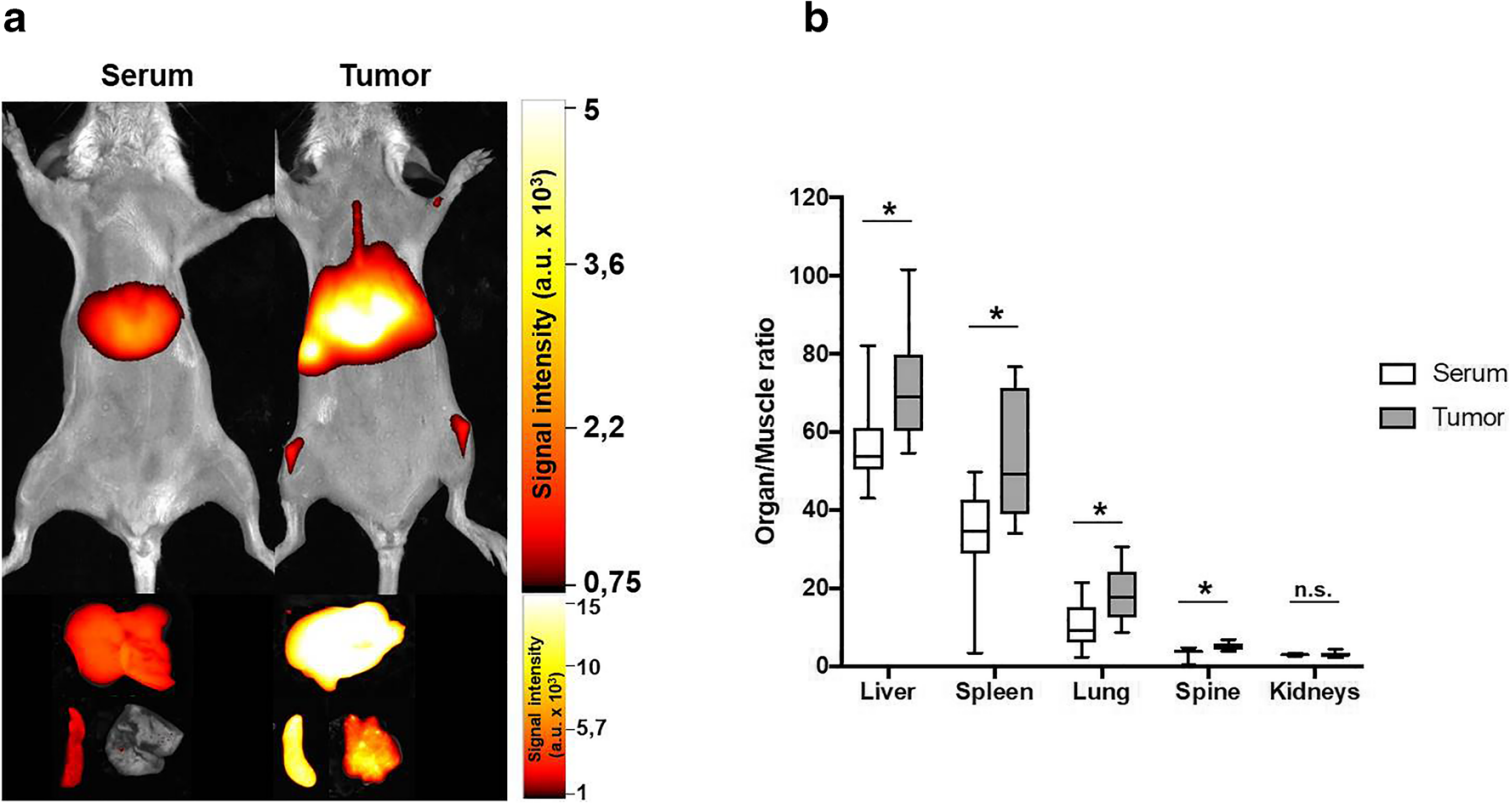
Comparison of the organ accumulation of extracellular vesicles after 24 h reveals a significant difference between extracellular vesicles from healthy mice and tumor cell–derived extracellular vesicles. **a** Exemplary *in* and *ex vivo* data. **b** Results of at least three different experiments.

When compared to the distribution of extracellular vesicles from healthy BALB/c mice, accumulation of tumor cell–derived extracellular vesicles was significantly increased in lung, liver, spleen, and spine, whereas no differences were observed for the other analyzed organs (kidneys, pancreas, brain, and heart). Mean intensities [a.u.] of tumor extracellular vesicles *vs.* serum extracellular vesicles were lung 18.6 ± 6.6 *vs.* 10.4 ± 6.1, *p* = 0.01; liver 72.2 ± 14 *vs.* 56.5 ± 11.8, *p* = 0.02; spine 5.1 ± 1 *vs.* 3.5 ± 1.3, *p* < 0.01; spleen 53.4 ± 15.6 *vs.* 32.8 ± 13.9, *p* < 0.01; kidneys 3 ± 0.6 *vs.* 3 ± 0.3, *p* = 0.98 (Fig. 4).

### Changes in the Immune Cell Composition

**Fig. 5:**
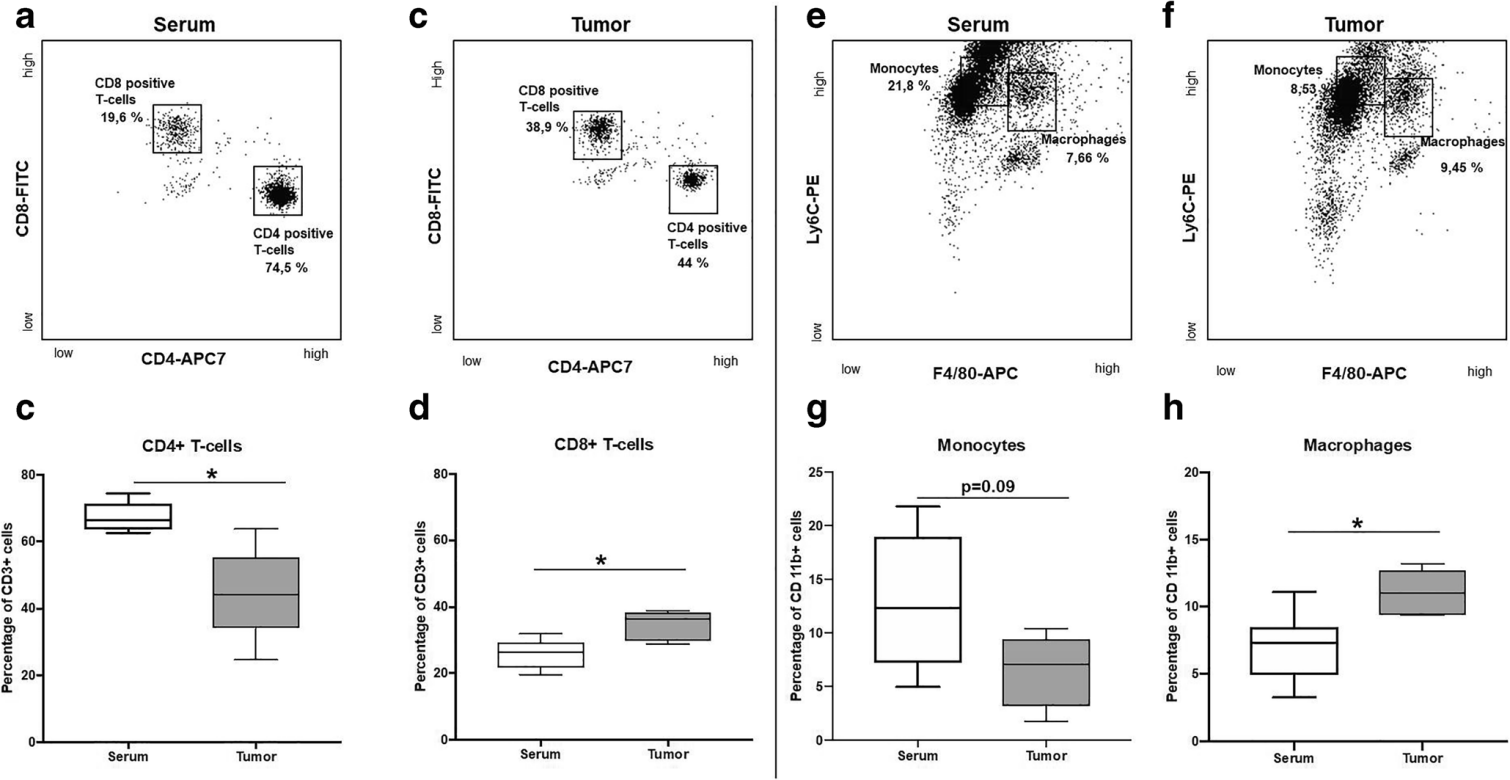
Results of the flow cytrometry experiments. **a**–**d** Flow cytometry plots and the corresponding results of the T cells analysis **c** reveals a decrease in T-helper cells (*p* < 0.05) and **d** an increase in cytotoxic T cells (*p* < 0.05). **e**–**h** Analysis of the CD11b positive cells showed G a trend towards a decrease in monocytes (*p* = 0.08) and H an increase in macrophages (*p* < 0.05).

Differences in the immune cell composition of the lungs 24 h after a single injection of 100 μg extracellular vesicles were identified between tumor cell–derived extracellular vesicles and those from the serum of healthy BALB/c mice. A trend towards a reduction in T cell abundance, with a lower percentage of CD4^+^ T cells after injection of tumor cell–derived extracellular vesicles (67 % *vs.* 44.5 % after injection of serum extracellular vesicles, *p* < 0.05) and a higher percentage of CD8^+^ T cells (35 % *vs.* 26 %, tumor cell–derived *vs.* serum-derived, *p* < 0.05) was observed. Furthermore, a trend towards decrease in monocytes (13 % *vs.* 6 %, tumor cell–derived *vs.* serum-derived, *p* = 0.09) and an increase in mature macrophages (7 % *vs.* 11 %, tumor cell–derived *vs.* serum-derived, *p* < 0.05) was detected (Fig. 5).

## Discussion

The extent of metastatic disease strongly determines the prognosis of cancer patients. The challenge to understand the process of metastatic spread with the potential to interfere is thus of paramount importance in cancer research.

Among the initial steps in the development of metastasis is premetastatic tissue priming, the induction of a premetastatic niche in the target tissue of metastasis.

The premetastatic niche, as a supportive and receptive tissue microenvironment, relies on three factors—the local stromal microenvironment, tumor-mobilized bone marrow–derived cells, and tumor-derived factors [17]. Tumor-derived factors are molecules like vascular cell adhesion protein 1 that mediates pro-metastatic tumor-stromal interactions and aids in the formation of metastasis [18], or heat shock proteins HSP70 and 90 that are known to enhance migration and invasion, as well as to participate in the establishment of an immunosuppressive environment [19].

Extracellular vesicles are currently being discussed as possible key players in metastatic spread, functioning as a transport medium for mediators of premetastatic niche induction [17].

Driven by the question if the pattern of extracellular vesicle distribution, accumulation, and uptake is an additional factor in the process of premetastatic tissue priming, we established a protocol for the isolation and labeling of extracellular vesicles, enabling *in vivo* tracking by optical imaging.

We characterized the extracellular vesicles from tumor cells and healthy mouse serum for their size and surface markers and analyzed the proteome cargo in search of proteins relevant for premetastatic tissue priming.

We followed the isolated and labeled extracellular vesicles *in vivo*, recording the organ-specific distribution of tumor cell–derived extracellular vesicles as compared to controls and assessed the target tissue of metastasis for extracellular vesicles-related changes of the immune cell composition. After labeling with the near-infrared dye DiR, extracellular vesicles can afterwards be visualized *in vivo* using fluorescence reflectance imaging [20]. This approach enables to track these microvesicles without changing their characteristics and membrane components, which is crucial for their distribution and the subsequent site of accumulation. DiR is a lipophilic dye that only exhibits a strong fluorescence signal when incorporated into a lipid membrane, so there is little background to the extracellular vesicles’ signal by free dye molecules [21]. Furthermore, labeling extracellular vesicles with DiR does not have effects on the vesicle morphology or integrity [22, 23]. A comparable lipophilic dye, DiD, was already successfully used to assess the *in vivo* distribution of extracellular vesicles [24].

Mass spectrometry revealed marked differences between extracellular vesicles from healthy mouse serum as well as tumor cell–derived extracellular vesicles. Mainly, three groups of proteins have been described in extracellular vesicles: antigen-binding proteins such as heatshock proteins and MHC proteins class I/II, proteins involved in signal transduction (*e.g.*, annexins, tetraspanins, integrins), and cytoskeletal proteins (*e.g.*, actin, ezrin, tubulin) [25]. In our study, tumor cell–derived extracellular vesicles contained a higher amount of dedicated heatshock proteins (Hsp 70 and 90), leukocyte adhesion molecules and molecules involved in leukodiapedesis (VCAM-1, Talin-1, integrin beta 2), proteins modulating endothelial barrier function and permeability (Ras-related proteins Ib, 2, 7a), higher amounts of inflammatory proteases such as serin proteases (whereas extracellular vesicles from healthy serum contained higher amounts of serin protease inhibitors), extracellular matrix proteins involved in invasion and metastasis (SPARC, syndecan-4), markers of proliferation (Mib1, CTGF) and general markers of malignancy (moesin, CTGF). Correspondingly, other studies also found extracellular vesicles from 4T1 tumor cells as compared to those from 67NR tumor cells with a lower malignant potential, to contain higher amounts of integrin beta-1, an important factor in migration, and L-lactate dehydrogenase (Ldha). An enrichment of plasminogen activator, promoting extracellular matrix degradation and invasiveness, was previously reported. [26]

A network analysis of the abundant proteins in tumor cell extracellular vesicles (Fig. 2b, Supplementary Table S10) revealed a large protein cluster surrounding T-complex 1 subunit eta and cell cycle and apoptosis regulator protein 2 with branches to tumor-relevant proteins such as syndecan-4 (Fig. 2b, red). In summary, our analyses of the extracellular vesicle cargo suggests that tumor cell–derived extracellular vesicles carry a variety of specific proteins, which are also involved in reformation of the host immune system towards a tumor-permissive microenvironment including alteration of endothelial function and recruitment and proliferation of pro-tumorigenic immune cells.

Following *in vitro* and *ex vivo* analyses, *in vivo* experiments were performed to assess the kinetics of extracellular vesicle distribution and accumulation. We observed a first, unspecific pass of the extracellular vesicles through the measurable organs *via* the blood stream, followed by a specific accumulation of tumor cell extracellular vesicles in selected organs. The extracellular vesicles from healthy mouse serum did not show a conclusive, specific distribution, following the initial first pass and accumulated in liver and spleen, as might be expected for subcellular vesicles of a certain size [22].

To minimize the risk of dye transfer from labeled extracellular vesicles, increasing unspecific background signal and the results from *in vivo* distribution studies, further detailed analyses were performed at 24 h after extracellular vesicle application [23, 27, 28]. At 24 h already, and more pronounced at 48 h after extracellular vesicle application, the two extracellular vesicle populations showed a different distribution pattern. *In vivo* imaging, as well as *ex vivo* biodistribution analysis of selected organs revealed an increased accumulation of tumor cell–derived extracellular vesicles in the lung, the primary target organ of metastasis in the 4T1 model [29], as compared to serum-derived extracellular vesicles from healthy mice. This effect could also be observed in the liver and the spine, which are also typical sites of distant metastasis [30]; the effect in the liver was partially masked by the high background accumulation, the effect in the bone was barely detectable due to the limitations of optical imaging of bone marrow processes. The results indicated a specific accumulation in organs of subsequent metastasis, which is in line with the results from a previous study [24].

The comparison of tumor cell–derived extracellular vesicles with a heterogeneous population of extracellular vesicles from healthy mouse serum, instead of liposomes as in numerous comparable studies [24], takes into account potential biological effects of the physiological extracellular vesicle population and immunological reactions to an increase in the extracellular vesicle load. The additional effects, observed in this study, especially the changes in the immune cell population of lung tissue can thus be attributed to the tumor cell–derived extracellular vesicles and their active cargo.

However, future studies will have to investigate the potential contribution from extracellular vesicles, shed by tumor-associated immune cells in the immediate tumor microenvironment or systemically in response to tumor-secreted factors.

We further examined the biological effects of tumor cell–derived extracellular vesicles in the lungs of extracellular vesicle-treated animals. Twenty-four hours after a single administration of extracellular vesicles already, the immune cell composition of the host animals showed measurable changes. We observed a numerical decrease of CD4^+^ T cells, which inhibit pro-tumor regulatory T cells as well as anti-tumor natural killer cells. Regarding these seemingly contradictory effects of CD4^+^ T cells, their prognostic value remains unclear [30, 31]. The reason for the simultaneous increase of CD8^+^ T cells remains similarly unclear. However, as cytotoxic T cells exhibit a potent anti-tumor effect, their increase might be a response to initial changes of the immune cell composition towards a premetastatic niche [32]. Our observations seem contradictory to the study by Wen et al. [24], who did not observe significant numerical changes in the pulmonary T cell population after administration of extracellular vesicles. However, the treatment regimen employed in their study with repeated applications of extracellular vesicles over several days might lead to further changes in the pulmonary immune cell population, partially reverting the early effects.

The relative decrease of monocytes after treatment with tumor cell–derived extracellular vesicles and the simultaneous increase of macrophages might be due to promotion of an accelerated maturation of monocytes to tumor-associated macrophages. The increase of tumor-associated macrophages is a crucial step not only in the progression of the primary tumor, but also the development of distant metastases [33]. Thus, their local increase after injection of tumor cell–derived extracellular vesicles is an indicator for early, extracellular vesicle-driven changes in the immune cell composition towards a tumor-receptive environment.

## Limitations

A limitation of our study is the route of extracellular vesicle application. In order to label the vesicles for subsequent tracking, isolation was necessary. The route of administration *via* the tail vein and the consecutive, rapid distribution in the blood pool might not in all aspects represent the “natural” route, extracellular vesicles reach systemic distribution from the primary tumor.

In follow-up projects, extracellular vesicles from tumor types with different metastatic patterns will be evaluated, based on the presented study to answer the question, immediately provoked by our results: does the distribution pattern fully explain organotropism of specific tumor types or are additional factors required? We believe to have given a sound basis for these future studies.

## Conclusions

In conclusion, tracking of DiR-labeled extracellular vesicles *in vivo* using fluorescence reflectance imaging enables the *in vivo* visualization of their distribution and specific accumulation in target organs of subsequent metastasis. The pattern of this distribution is different between extracellular vesicles, derived from tumor cells and bulk vesicles from healthy controls and mirrors the potential pattern of metastasis. Tumor cell–derived extracellular vesicles contain a variety of proteins with the potential to establish a tumor-receptive microenvironment in potential target organs of metastasis. The observed alterations of the pulmonary immune cell population suggest that the establishment of an immunosuppressive environment as described for the premetastatic niche follows this initial phase of immune cell rearrangement. Our work further emphasizes the role of extracellular vesicles as potential mediators of tumor spread but, with a view to comparable studies, also hints towards a specific kinetic course of metastatic spread and the preceding microenvironmental changes. This time line as well as the distribution pattern as a potential factor of organotropism should be the focus of further cancer research. The suggested *in vivo* imaging approach, in combination with proteome and cell population analyses can serve as a tool for further research in the mechanism of systemic tumor spread.

## Electronic Supplementary Material

ESM 1 (DOCX 273 kb)

ESM 2 (XLSX 2616 kb)
