# Supplementary material for: Tracking of Tumor Cell–Derived Extracellular Vesicles In Vivo Reveals a Specific Distribution Pattern with Consecutive Biological Effects on Target Sites of Metastasis
Source: Mol Imaging Biol. 2020 Jul 31;22(6):1501–10. doi: 10.1007/s11307-020-01521-9 (PMC7666295; doi:10.1007/s11307-020-01521-9)

**Supplementary Figure 1.** Experimental design of the study.


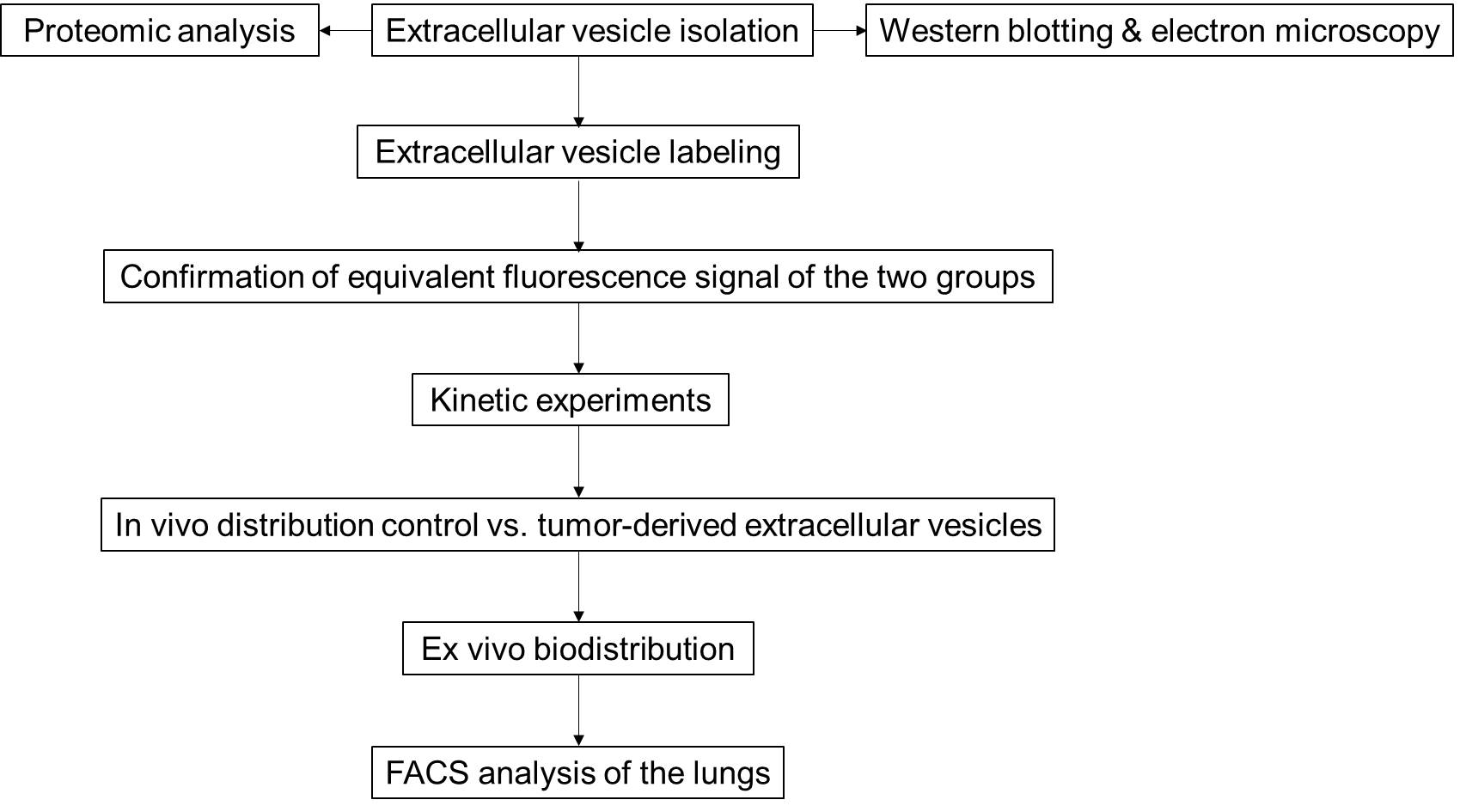


**Supplementary Figure 2.** Work flow of extracellular vesicle isolation and labeling. Adapted from ^10^.


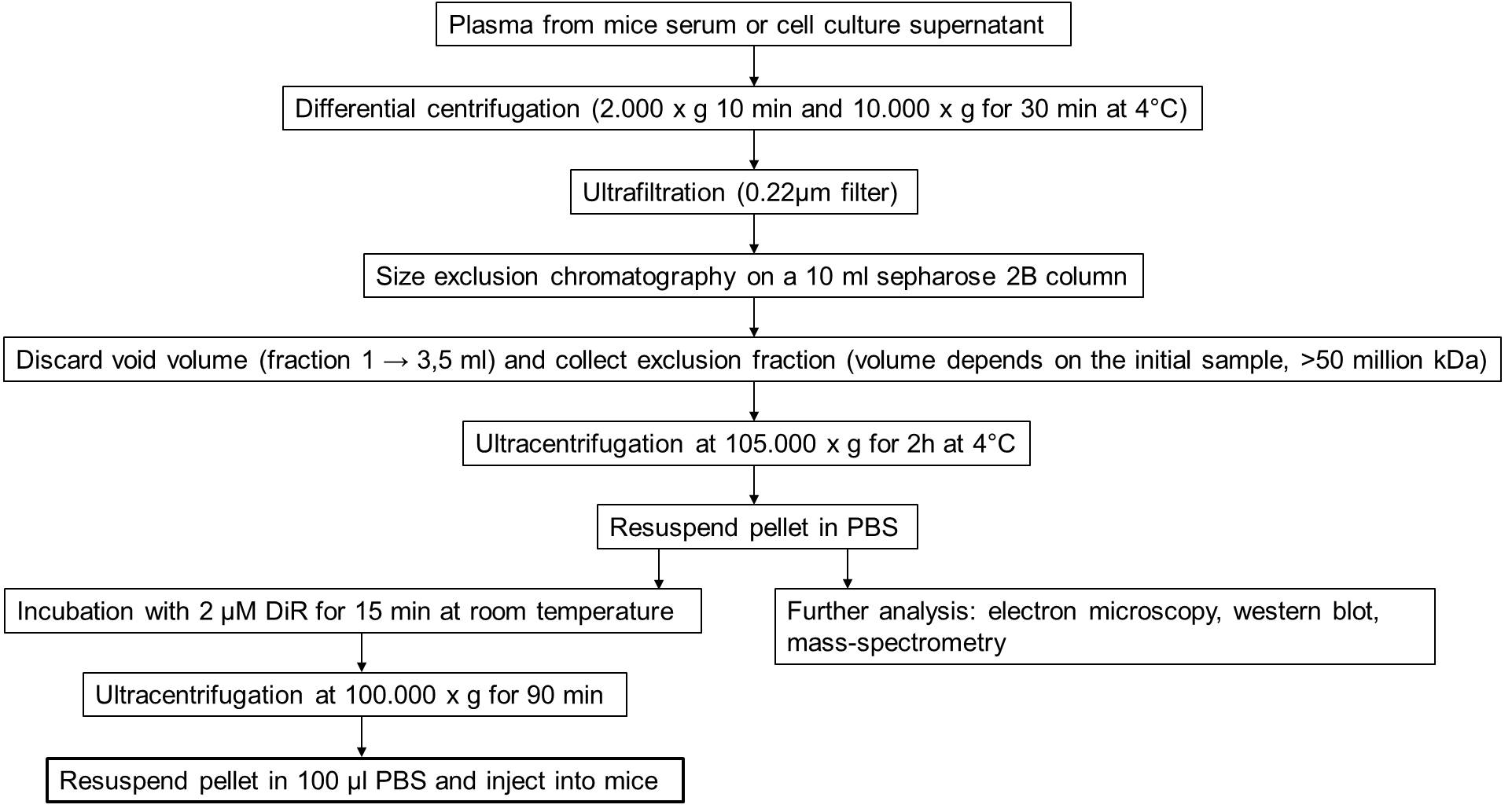

Supplement: Supplementary file 1 — (DOCX 273 kb) [file 11307_2020_1521_MOESM1_ESM.docx]
